# Are Malaysian Youths Overdependent on the Internet?: A Narrative Review

**DOI:** 10.3389/fpsyt.2021.710790

**Published:** 2021-08-13

**Authors:** Nik Ruzyanei Nik Jaafar, Norharlina Bahar, Normala Ibrahim, Azlin Baharudin, Wan Salwina Wan Ismail, Su Tein Sim, Melisa Abdul Aziz, Kit-Aun Tan

**Affiliations:** ^1^Department of Psychiatry, Faculty of Medicine, Universiti Kebangsaan Malaysia, Kuala Lumpur, Malaysia; ^2^Malaysian Society of Internet Addiction Prevention, Serdang, Malaysia; ^3^Department of Psychiatry, Prince Court Medical Center, Kuala Lumpur, Malaysia; ^4^Department of Psychiatry, Faculty of Medicine and Health Sciences, Universiti Putra Malaysia, Serdang, Malaysia; ^5^AHL Specialist Clinic, Petaling Jaya, Malaysia; ^6^Department of Psychiatry and Mental Health, Hospital Ampang, Kuala Lumpur, Malaysia

**Keywords:** internet overdependence, youths, Malaysian, narrative review, prevention, intervention

## Abstract

Overdependence on the internet is a grave concern that has enveloped Malaysian youths which could lead to a variety of sequelae. This narrative review aims to determine the definition of internet overdependence and its associated factors, as well as the potential preventive and treatment strategies for internet overdependence. From the literature, internet overdependence is regarded as a 3-factor model encompassing salience, self-control failure, and serious consequences. Sociodemographic factors such as age, gender, and ethnicity, as well as psychosocial factors such as depression, anxiety, stress, and loneliness, were found to be associated with internet overdependence among Malaysian youths. A multimodal treatment approach is recommended by the implementation of various types of treatments, integrating disciplines such as pharmacology, psychotherapy, and family counseling. Despite various terminologies being used and unclear conceptualization of its nomenclature, overdependence on the internet is prevalent among youths in Malaysia. Future research should go toward establishing a clear definition of its terminology and attaining more robust evidence on treatment strategies.

## Introduction

The good and the bad of the internet has been a continuous debate—it's penetration rate in youths is of particular concern. Malaysia recorded a notable increase in national internet usage from 76.9 to 88.7%, in a 4-year period, with 85.9% of them being individuals aged 44 and below (1). Although Malaysia has good nationwide internet connectivity, and Malaysians in general enjoy equal opportunity for internet access, overdependence on the internet is a grave concern that has enveloped Malaysian youths which could lead to a variety of sequelae.

There exists a rich collection of local internet addiction studies. Of these, a handful of internet overdependence studies targeting primary, secondary school students, college, and university students were reported (2–9). Often, the emphasis of these studies was placed on the variables that were linked to internet overdependence, however, less attention has been paid to researching its consequences and implications. The internet users' behavior in Malaysia was reported to be socially motivated as they were more inclined to engage in leisurely online activities that were combined with social networking (1, 10). Notably, there is growing concern over the emergence of internet overdependence as a public health issue.

Therefore, the aims of this review are to describe what constitutes internet overdependence and to examine its associated factors with reference to Malaysian youths. We conclude the review by suggesting strategies that could be implemented to curb overdependent usage of the internet among young people in Malaysia.

### Definition of Internet Overdependence

Driven by its triple A engines—anonymity, accessibility, and affordability, the internet provides a new social milieu for social interaction (11). Under the “addiction” umbrella, maladaptive patterns of internet use have garnered much attention in terms of research. Some researchers prefer to define such maladaptive patterns as *internet addiction* (12), others prefer *problematic internet use* (13), and still others prefer *internet dependency* (14). Despite differences in terminology, they are perhaps theoretically identical and have often been used interchangeably. For ease of discussion, we use the term *internet overdependence* in this review paper.

The term over-dependence was first used by Korea's institutions such as National Information Society Agency and Internet Addiction Prevention Center in an attempt to facilitate research on problematic Internet use in local setting. To better reflect the changing digital media environment in which smart devices are frequently used in accessing the internet, it appears that the term *over-dependence* may have been more appropriate, as it avoids over-pathologizing those who excessively used smart media, recognizing internet devices and smart media are integral to their everyday lives (15).

As a behavioral addiction, internet overdependence can lead to the erosion of physical, social, and psychological functioning (16, 17). All forms of addictions, whether chemical or behavioral, share certain characteristics. These include salience, compulsive use (loss of control), mood modification, and alleviation of distress, tolerance, and withdrawal, as well as continuation despite negative consequences (18). Internet Addiction Disorder is a concept first proposed by Goldberg (19) in 1996 as an analogy to substance dependence, but Young et al. (20) redefined this condition as an impulse-control disorder not involving an intoxicant. However, researchers also argued that the word “addiction” was disparaging and would lead to patient alienation (21). Therefore, “overdependence” was suggested to replace “addiction.” According to the 2016 Survey on Internet Usage (22), internet overdependence consists of three factors: (a) salience—a condition in which internet use becomes the most salient and important activity in one's daily life, (b) self-control failure—a condition in which one is not able to control themselves on internet use in accordance with self-set goals, and (c) serious consequences—a condition in which one experiences negative consequences in physical, psychological, and social aspects due to problematic internet use.

Evidence shows that the neural mechanisms of internet overdependence resemble those of substance use disorder. It is known that substance addictions activate the reward center in the brain by increasing dopamine release, along with opiates and other neurochemicals (23, 24). Over time, this would produce a tolerance effect whereby the receptors need stimulation of the reward center to produce the same level of pleasure sensation. The tolerance effect would eventually be followed by the development of characteristic behavior patterns to avoid withdrawal. Internet use would also lead specifically to dopamine release in the nucleus accumbens which is part of the reward center (25).

Internet overdependence was found to be associated with pre-existing psychological issues such as depression, loneliness, and anxiety in past studies (10, 26–28). Individuals who develop internet overdependence often use the internet as a coping mechanism to escape from real-life problems (29). This vicious cycle, where individuals with psychological issues avoid social interaction and try to escape from the problems through internet use, can further worsen their psychological disturbances. This process is similar to the drug or alcohol abuser who has underlying psychological issues and tries to self-medicate (29).

### Factors Associated With Internet Overdependence

#### Sociodemographic Factors: Age, Gender, and Ethnicity

There is a wide variation in the prevalence rates of internet overdependence among youths in Malaysia. The prevalence rates of such behavioral addiction ranges from 7.8 to 60.7% (5–8, 30, 31) in cross-sectional samples involving youths from Malaysian public universities. This disparity is similarly reported in other Asian countries in part due to cultural and social differences within each nation and across the regions (32). Another possibility lies on other factors such as internet accessibility as well as government policies which might contribute to internet overdependence problems across the regions (33). The use of different assessment tools and different languages could contribute to the variability in the reported prevalence rates. It appears that the Internet Addiction Test, in the English or Malay versions, was widely used in local studies from Malaysia [e.g., (34)], whereas the Revised Chen Internet Addiction Scale and other language versions of the Internet Addiction Test were widely used in local studies from other Asian regions [e.g., (4)].

As far as age is concerned, while there is inadequate evidence to suggest that internet overdependence might represent a problem specific to young people, this issue is relatively more prevalent in youths (35–37) compared to other age groups. In an internet usage survey, 56.5% of internet users in Malaysia were below 29 years old (38). Being born in the digital era, the current generation of youths residing in urban areas in Malaysia is reportedly exposed to the internet since the age of 5 years old (39). According to the technological determinism theory, early internet exposure has been recognized as an important factor contributing to internet overdependence (40) among college and university students (9, 31, 37) as they rely enormously on the internet for academic purposes (9, 41, 42). As a consequence, these youths spend a considerable time online and stay online longer than they intend to. Frequent internet use and lengthy online duration were significant factors contributing to problematic internet use among youths in Malaysia (3, 31).

With respect to gender differences, in line with studies abroad (43, 44), internet overdependence was more prevalent in male than in female internet users in Malaysia (3, 31, 38, 39, 45, 46). However, gender may not be as significant a role as was previously thought. Internet overdependence was found to be an emerging public health problem in Vietnamese youths and it was found that young women were equally as vulnerable as men (33). It has been proposed that the motivation of its use differs across the genders. It is reported that women use the internet for social interaction, information-gathering, and shopping, whereas men surf the internet for adult sites (43, 45), gaming (47, 48), and gambling (47). However, there were no gender differences in many aspects including duration, frequency, access to internet, and experience of internet use locally and universally (33, 45, 46).

Ethnic differences in terms of computer ownership, online duration, frequency, and experience of internet use were reported in one local survey study involving urban youths (49). Participants that belonged to the Chinese ethnic group were found to own more computers, spent longer time online, and used the internet more frequently as compared to other ethnic groups (49). However, this finding was not replicated in other local studies. The associations between internet addiction and subjective wellbeing were found to differ significantly by ethnic groups in Malaysia (50). Taken together, these findings shed light on the importance of future cross cultural-studies on internet overdependence as the findings may be confounded by educational and socio-economic status.

#### Psychosocial Factors and Sociocultural Influence

Motivations for going online were for entertainment purposes, online gaming, and educational-related activities. Social networking was found to be one of the main reasons for going online. In support of this premise, Omar et al. (51) surveyed a total of 400 youths across four zones in Malaysia and found that the principal idea of internet usage revolves around one's social relationship which in turn leads to the promotion of subjective wellbeing. The internet could be utilized to promote youth wellbeing but its usage must be monitored to prevent overdependence.

Internet overdependence was found to be significantly associated with poor social interaction and psychological distress (5, 6). It has been suggested that it could displace valuable time usually spent with family and friends (5). Problematic internet use has also been associated with psychological attributes such as depression, anxiety, stress, and loneliness among Malaysian youths (7, 10, 52–56). In a local study involving university undergraduates, problematic internet users were found to have higher levels of depression compared to non-problematic users (55). It appears that university students went online when they were depressed and used the internet to escape from the problems they faced. Although the internet reduced their unhappiness and provided emotional support, they became anxious when they were not connected online (57, 58).

Besides depression and general anxiety, previous studies focusing on other psychiatric domains in relation to internet overdependence were also conducted. NOMOPHOBIA or NO MObile PHone PhoBIA is the term describing one's psychological fear of being detached from mobile phone connectivity, as stated in the *Diagnostic and Statistical Manual-4* for specific phobia (59). It has not been well-explored in Malaysia but a recent study found that nomophobia had a strong significant positive association with internet overdependence (60). Another psychiatric domain, i.e., Attention Deficit Hyperactivity Disorder symptoms was also found to be significantly associated with problematic use of internet among Malaysian youths (3). Social phobia was found to be associated with internet overdependence in a local university student sample (61). There are local studies documenting that prolonged smartphone usage could result in poor sleep quality, daytime tiredness, and internet overdependence (8, 62).

Comparing this globally, a similar relationship between internet overdependence and poor psychological well-being has been reported regardless of the developmental status of the country (32, 33, 63, 64). Heightened psychological arousal associated with excessive internet use has been suggested to underlie the resultant health problems (32). Interestingly, Tran et al. (33) reported that Vietnamese youths with internet addiction derived interpersonal influences on their lifestyles and behaviors from online relationships—an area worth examining further—particularly in understanding how this impacts their psychological health.

From the sociocultural aspect, the internet shortens the communication lines between individuals which helps to strengthen and expedite cultural exchanges across the globe (65). Perhaps, the globalization of culture through the internet lends some insight to almost universal or culture-free pattern and psychosocial risk factors observed in internet overdependence. In contrast, persistent discrepancies exist in worldwide internet consumption which may reflect culture-specific communication preferences. This may necessitate cultural adaptation in addressing the sequelae of internet overdependence. Also, the influences of politics and economy on Internet usage may be more pronounced in developing nations like Malaysia and Southeast Asia than in highly developed countries (65).

### Preventive and Treatment Strategies

In general, preventive and treatment strategies for internet overdependence have not been extensively reported globally as most prior studies were devoted to investigate its risk factors and complications (66). Similarly, in Malaysia, there were numerous studies examining the prevalence, vulnerabilities, and risk factors of internet overdependence but studies investigating the treatment strategies for internet overdependence were scarce (9, 31, 37). This is due to the lack of conceptualization of the term used to define overdependence on the internet as it has yet to be recognized as a specific disorder in the *Diagnostic and Statistical Manual-5* diagnostic criteria. Therefore, the lack of diagnostic validity makes planning for specific treatment a challenge. Research findings reporting treatment efficacy were inconclusive because of these conceptual issues (67).

Findings from previous studies have recommended that strategies to curb overdependence on the internet should focus on preventive measures rather than specific focused treatment (68, 69). Specific focused treatment is important if there are co-morbid illnesses such as depression or anxiety. Preventive measures to identify populations at-risk should address the predisposing or vulnerability factors which contribute to the development of internet overdependence. Efforts should include looking into the roles of family, schools, and community as these social domains are central to the development of morals and values during formative years among youths (70). A global preventive level that focuses on primary prevention, as well as selective level of prevention that focuses on identifiable risk factors such as psychopathology and personality are recommended. Prevention measures should target children, adolescents, and young adults as these groups were found to be at higher risk for internet overdependence as compared to the other age groups (70).

There is a general consensus that abstinence from problematic applications as well as controlled and balanced internet usage should be achieved rather than total abstinence from the internet (71). A multimodal treatment approach is characterized by the implementation of various types of treatment which should include disciplines such as pharmacology, psychotherapy, and family counseling simultaneously or sequentially (17). The uses of selective serotonin reuptake inhibitors, naltrexone, mood stabilizers, or antipsychotics in individuals with internet overdependence are also suggested (27). However, the literature review in this area is still inconclusive and more robust evidence is required.

Understanding the neurobiological process of the condition may help to alleviate the condition as suggested by recent advances in treating internet overdependence. Li et al. (72) reviewed an exercise-based intervention in mitigating such condition. It was reported that exercise-based intervention can stimulate the neurogenesis of the hippocampus *via* regulation of the level of neurotrophic factors and neurotransmitters. This in turn would regulate the reward mechanism in the brain. Past studies have shown the benefits of exercise in countering internet addiction. Zhang (73) reported that exercise-based intervention had psychological benefits for internet addiction as an alternative or adjunct therapy as it improved co-morbid symptoms such as depression and anxiety. In one study, exercise has shown to reduce the severity of the condition and the time spent online (74). Meta-analytic findings also reveal that sports interventions could significantly reduce internet addiction (75).

Another mode of treatment that has been extensively reviewed for substance and behavioral addiction is neuromodulation. Neuromodulation targets on the neurocircuits abnormalities in the brain. Among these techniques are transcranial direct current stimulation (tDCS) and (repetitive) transcranial magnetic stimulation (rTMS). Past studies have investigated the efficacy of neuromodulation techniques and their effects on cognitive processes related to addiction (76–80). tDCS had shown significant effects on gaming time and internet scores reduction in a sample of online gamers (81). On the other hand, rTMS had demonstrated significant effects in deducing cue prompted cravings among subjects (82), but this finding was not observed elsewhere (83). One of the factors that may result in the contradicting outcomes in the neuromodulation application for internet related addiction is the heterogeneity of the condition with diverse individual differences that underlies the abnormalities of the brain (84). This offers a highly promising area for further development in research and treatment. Therefore, it adds on to more therapeutic options in internet-related disorders.

In terms of psychotherapy, motivational interviewing, cognitive-behavioral therapy, reality therapy, as well as acceptance and commitment therapy were found to reduce internet overdependence (17, 25, 85, 86). As mentioned above, physical exercise represents a promising treatment approach as it could compensate for the decreases in dopamine due to decreased online usage (17). In addition, sports exercise prescriptions used in the course of cognitive behavioral group therapy may enhance the effect of the intervention for internet overdependence (87).

The residential treatment approach in internet use related disorder has been discussed in some literature but there is inadequate scientific evidence in its clinical utility. One study in Korea has reported the effectiveness of a 12-day residential program comprising education and alternative recreational activities (88) and found that subjects improved significantly in self-reported and parents' scores in internet addiction scales. Subjects also showed significant changes in other measures such as depression, gaming time, self-control, and sense of well-being. Another residential study in Japan also reported significant results whereby adolescent subjects had reduced their time spent on online gaming (89). It appears that the residential treatment approach could assist in restricting access to the internet related devices. This has an advantage to separate them physically from the devices and acts *via* the detoxification method. This may serve as an opportunity to subjects to engage in intensive psychological treatments and help them to build interpersonal relationships thanks to the therapeutic component of this approach.

In Malaysia, the 1998 Communication and Multimedia Act provides a legal mandate to defend a free and open internet. This mandate is overseen by government agencies such as Malaysia Communications and Multimedia Commission, and Cybersecurity Malaysia which were established to promote and regulate the development of communications and multimedia activities and to strengthen cybersecurity (90, 91). With the establishment of National Cyber Security Agency, the existing cybersecurity activities are further coordinated to strengthen Malaysia's cyber-system (91). However, a national policy to regulate internet use particularly for youths is yet to be established. Specific regulation and strict enforcement (92) is timely given the rising numbers of young people with internet overdependence in Malaysia. The Malaysian government can also emulate countries like Japan and South Korea that limit internet access among underage users for a duration of time, at specific time frames. Furthermore, educational promotion about the consequences of the uncontrolled internet may be beneficial (93).

### Directions for Internet Overdependence Research

Internet overdependence has become an increasing public health concern but its evidence in preventive and treatment strategies is still lacking. Future internet overdependence research can focus on conducting a large-scale local prospective study integrating both preventive and treatment strategies. In addition, identification of neurobiological basis of internet overdependence would be a platform to design related biological treatment strategies for the condition. Subsequently, this will aid in the development of specific pharmacological treatment of internet overdependence.

## Conclusion

Despite various terminologies being used and unclear conceptualization of its nomenclature, overdependence on the internet is prevalent among youths in Malaysia. It is associated with certain modifiable risk and vulnerability factors. However, evidence on its preventive and treatment measures is lacking and inconclusive. Therefore, future research should go toward establishing a clear definition of its terminology and attaining more robust evidence on treatment strategies. In addition, the neurobiological etiology of this condition should be extensively studied in order to assist in the exploration of its therapeutic treatment modality in view of its nature, which differs from substance dependence.

## Author Contributions

NRNJ and NI were involved in the conception and design of the review. All authors contributed to earlier drafts of the manuscript, which was finalized by NRNJ, NI, and K-AT. All authors did the literature searches and screened articles for inclusion and approved the final version of the article.

## Conflict of Interest

The authors declare that the research was conducted in the absence of any commercial or financial relationships that could be construed as a potential conflict of interest.

## Publisher's Note

All claims expressed in this article are solely those of the authors and do not necessarily represent those of their affiliated organizations, or those of the publisher, the editors and the reviewers. Any product that may be evaluated in this article, or claim that may be made by its manufacturer, is not guaranteed or endorsed by the publisher.
